# Association between *RASSF1A* Promoter Methylation and Ovarian Cancer: A Meta-Analysis

**DOI:** 10.1371/journal.pone.0076787

**Published:** 2013-10-08

**Authors:** Hao Shi, Ya Li, Xiaozhong Wang, Cheng Lu, Lilan Yang, Changmei Gu, Jiaqiang Xiong, Yangxin Huang, Shixuan Wang, Meixia Lu

**Affiliations:** 1 Department of Epidemiology and Biostatistics, and the Ministry of Education Key Lab of Environment and Health, School of Public Health, Tongji Medical College, Huazhong University of Science and Technology, Wuhan, Hubei, China; 2 Department of Obstetrics and Gynecology, Tongji Hospital, Tongji Medical College, Huazhong University of Science and Technology, Wuhan, Hubei, China; 3 Department of Clinical Laboratory, The Second Affiliated Hospital of Nanchang University, Nanchang, Jiangxi, China; 4 Department of Anatomy, Medical College of Nanchang University, Nanchang, Jiangxi, China; 5 Department of Obstetrics and Gynecology, The First Affiliated Hospital of Nanchang University, Nanchang, Jiangxi, China; 6 Department of Epidemiology and Biostatistics, College of Public Health, University of South Florida, Tampa, Florida, United States of America; University of Louisville, United States of America

## Abstract

**Background:**

The RAS association domain family protein 1a gene (*RASSF1A*) is one of the tumor suppressor genes (*TSG*). Inactivation of *RASSF1A* is critical to the pathogenesis of cancer. Aberrant *TSG* methylation was considered an important epigenetic silencing mechanism in the progression of ovarian cancer. A number of studies have discussed association between *RASSF1A* promoter methylation and ovarian cancer. However, they were mostly based on a small number of samples and showed inconsist results, Therefore, we conducted a meta-analysis to better identify the association.

**Methods:**

Eligible studies were identified by searching the PubMed, EMBASE, Web of Science, and CNKI databases using a systematic searching strategy. We pooled the odds ratio (ORs) from individual studies using a fixed-effects model. We performed heterogeneity and publication bias analysis simultaneously.

**Results:**

Thirteen studies, with 763 ovarian cancer patients and 438 controls were included in the meta-analysis. The frequencies of *RASSF1A* promoter methylation ranged from 30% to 58% (median is 48%) in the cancer group and 0 to 21% (median is 0) in the control group. The frequencies of *RASSF1A* promoter methylation in the cancer group were significantly higher than those in the control group. The pooled odds ratio was 11.17 (95% CI = 7.51–16.61) in the cancer group versus the corresponding control group under the fixed-effects model.

**Conclusion:**

The results suggested that *RASSF1A* promoter methylation had a strong association with ovarian cancer.

## Introduction

Ovarian cancer is the fifth most common cause of cancer deaths in women and accounts for the highest tumor-related mortality of gynecologic malignancies. Approximately 1.5% of females suffer ovarian from cancer and most cases are diagnosed at late stage owing to the lack of effective early detection methods [1], [2]. Over 80% of patients with advanced ovarian cancer relapse [3]. While the 5-year survival rate is close to 30% [4].

Hypermethylation of the tumor suppressor gene (*TSG*) promoter can lead to gene inactivation, which is critical to the pathogenesis of cancers, and occurs always in the early stage of cancer development in many types of cancer including ovarian cancer [5], [6]. Some studies also showed that methylation of *TSG* was detected in tumor tissue and was associated with clinical features [7], [8]. The RAS association domain family protein 1A (*RASSF1A*) is a putative tumor suppressor gene located on 3 p21 spanning 11,000 bp, containing eight exons and two different promoters [9]. Epigenetic inactivation of the gene often resulted from the methylation of CpG islands in promoters [5]. An in vitro study showed that aberrant methylation was frequently observed in ovarian cancer cell lines [10]. These studies showed that the methylation of *RASSF1A* promoter may play an important role in the development of ovarian cancer.

Some studies have reported differences in the methylation frequencies of *RASSF1A* between cancer tissues and non-cancerous tissues. However, they were mostly based on a small number of samples and showed inconsist results. Therefore, we performed a meta-analysis to better identify the association between *RASSF1A* promoter methylation and ovarian cancer.

## Materials and Methods

### Search Strategy and Study Selection

**Figure 1:**
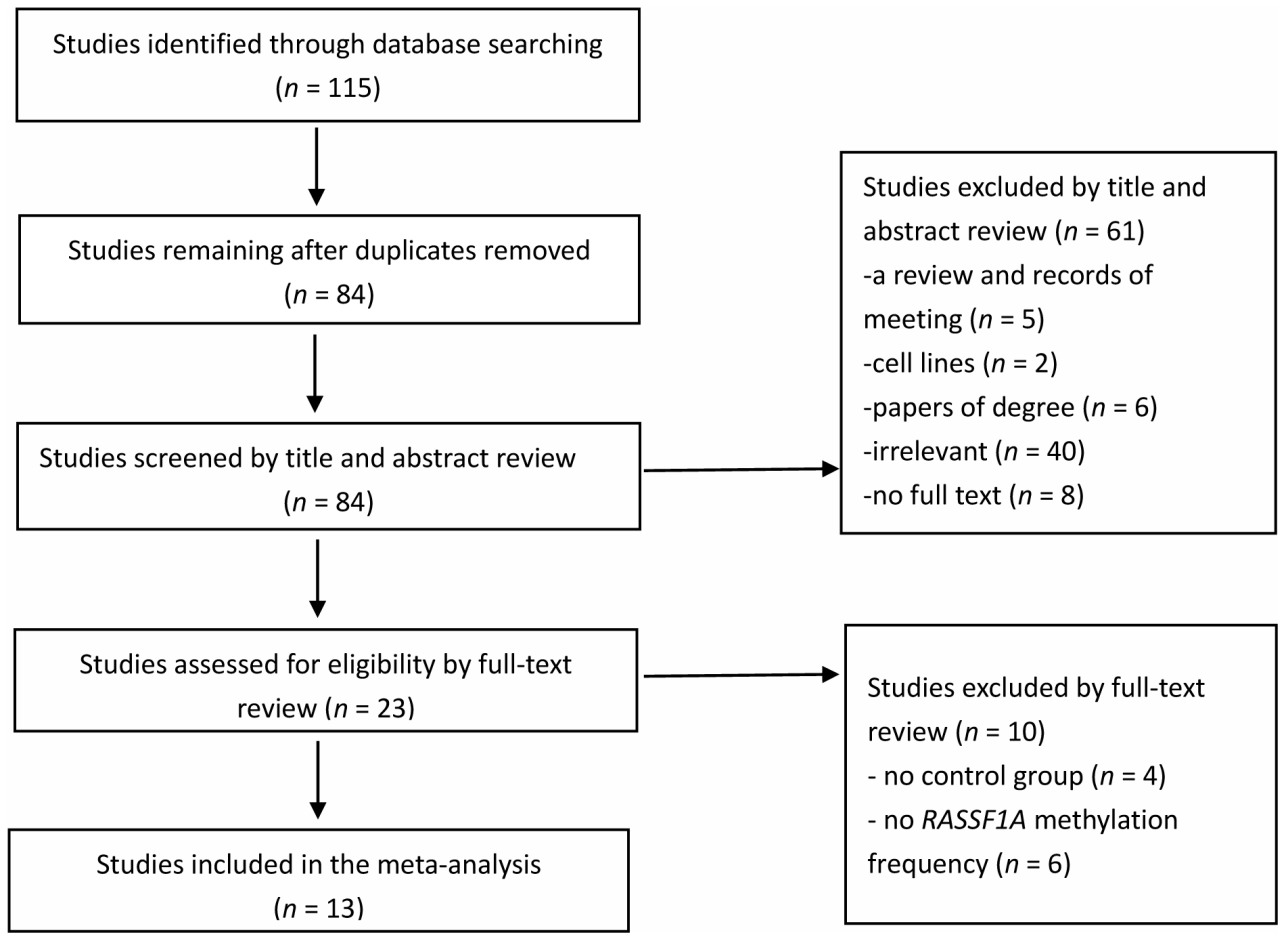
Selection of studies in the meta-analysis.

Online electronic databases (PubMed, EMBASE, Web of Science, and China National Knowledge Infrastructure (CNKI)) were searched for published studies up to June 1, 2013. The following search strategy was performed in PubMed: (ovarian OR ovary) AND (cancer OR carcinoma OR tumor) AND (*RASSF1A* methylation). The similar search strategy was used in other databases. The search was limited to human studies, without language restrictions. A study included in the meta-analysis had to meet the following criteria: 1) studies which evaluated the association of *RASSF1A* methylation with ovarian cancer, 2) a case-control study or one including case and control populations, 3) a study reporting the *RASSF1A* methylation frequency in case and control groups, 4) sample type limited to tissues. First, the title and abstract of studies from the initial search were evaluated according to the inclusion criteria. Then all potentially relevant studies were evaluated as full-text papers. If a study was published more than once, only the most complete and up-to-date information was included in the meta-analysis. Figure 1 presents detailed information on the study selection process. Finally, a total of 13 studies (PubMed 7, Web of Science 1, and CNKI 5) with 763 cases and 438 controls were included in our meta-analysis.

### Data Extraction and Quality Assessment

Three reviewers (Hao Shi, Ya Li, and Changmei Gu) independently reviewed the selected studies. The following information was extracted from these studies: first author’s name, year of publication, study population, sample size, age of women in the case group, control type, the status of the individuals in the control group, the number of individuals in the case and control groups, the measuring methods of methylation, and modulation frequencies of *RASSF1A* in the case and control groups. All the detailed information in the included studies was checked by four reviewers (Meixia Lu, Shixuan Wang, Xiaozhong Wang, and Yangxin Huang) as prescribed by the Cochrane Handbook for systematic reviews.

The quality of those studies was evaluated according to the Newcastle Ottawa Scale (NOS) (http://www.ohri.ca/programs/clinical_epidemiology/oxford.asp). The NOS has a maximum of nine ‘stars’ on items related to the selection of the study groups (four stars), the comparability of the groups (two stars) and the ascertainment of the outcome of interest (three stars). The studies were independently evaluated based on NOS by three reviewers (Cheng Lu, Lilan Yang, and Jiaqiang Xiong). Studies with quality scores greater than or equal to 5 were included.

### Statistical Analysis

To assess the strength of the association between *RASSF1A* methylation and ovarian cancer the pooled odds ratios (ORs) and their 95% confidence intervals (CIs) were calculated. The *x* ^2^-based Cochran Q statistic test and *I* ^2^ statistics were used to test the heterogeneity among the included studies [11]. The selected studies were considered to have a severe heterogeneity when the *I* ^2^ was greater than 50%. When *P*<0.05 for the Q statistic, the heterogeneity was considered significant and a random-effects model (the DerSimonian-Laird estimate) was used to calculate the pooled ORs. Otherwise, a fixed-effects model (the Mantel-Haenszel method) was applied. A meta-regression (restricted maximum-likelihood estimator method) was employed to explore the source of heterogeneity. Furthermore, a subgroup analysis was performed to evaluate the source of heterogeneity. The contribution of each study to the final results of the meta-analysis was evaluated according to sensitivity analysis. We also used the Peters test, the Begg’s rank correlation method [12] and a funnel plot for Egger’s test [13] to assess publication bias. The fail-safe number [14] was also employed to assess publication bias. In our study, all the *p* values were two sides with a significant level at 0.05. When the individual studies have cells with zero counts, the default is to add 0.5 to all zero counts in the Meta package. All statistical analyses were performed with the Meta package [15] (version 2.2-1) in R (version 3.00; http://www.r-project.org/).

## Results

### Study Characteristics and Quality Assessment

A total of thirteen studies with 763 cases and 438 controls was included in the meta-analysis. 115 studies were initially identified by searching the electronic databases. 84 potentially relevant studies were retrieved for further evaluation after removing 31 duplicated articles. Based on their titles and abstracts, 61 studies were excluded (5 reviews or meeting reviews, 2 cell lines, 6 thesis papers, 40 irrelevant articles, and 8 papers that did not have full text versions). Four studies without a control group and six studies without *RASSF1A* methylation data were excluded from full-text review. The studies were published between 2001 and 2012, and covered from 20 to 110 cases. Finally, 13 studies were included in our meta- analysis. Eight studies were of Asian subjects and five studies were of Caucasian subjects. Six studies analyzed methylation of *RASSF1A* promoter CpG islands [16]–[21] and seven studies analyzed *RASSF1A* promoter methylation [22]–[28]. The case group consisted of cancer tissues form ovarian cancer patients. The control group included adjacent tissues (AT) from ovarian cancer patients, ovarian tissue from benign ovarian disease patients (BOT), and normal ovarian tissue from cancer-free patients or healthy people (NT). Among the 13 included studies, 11 studies used methylation-specific polymerase chain reaction (MSP), 1 study used methylation-specific multiplex ligation-dependent probe amplification (MS-MLPA) and 1 study used bisulfite sequencing PCR (BSP) to explore *RASSF1A* methylation in ovarian cancer and control. Characteristics of the 13 studies are presented in Table 1.

**Table 1:** Characteristics of studies included in the study characteristics of included studies.

				Case		Control			
Author	Year	Country	Age (y)	M	U	M	U	Method	Control type
Ho [22]	2012	Taiwan	NA	47	63	0	29	MS-MLPA	BOT
Montavon [16]	2012	Australia	59.0	41	38	2	10	MSP	BOT
Bhagat [23]	2012	India	51.8 (22–71)	50	36	7	12	MSP	BOT
						0	15		NT
He [24]	2011	China	NA	16	26	0	19	MSP	BOT
						0	11		NT
Chen [17]	2010	China	44.3 (23–68)	36	26	13	38	MSP	BOT
						0	20		NT
Shen [25]	2008	China	52.8 (33–76)	31	32	1	19	MSP	NT
						1	9		AT
Wu [18]	2007	Norway	NA	23	24	0	4	MSP	BOT
Li [26]	2007	China	52.0 (36–89)	27	40	0	40	MSP	BOT
Makarla [27]	2005	USA	51.5 (20–86)	7	16	0	23	MSP	BOT
						2	14		NT
Ma [19]	2005	China	NA	42	38	0	80	MSP	AT
Ibanez [20]	2004	USA	62.0	16	19	0	10	MSP	BOT
						0	10		NT
Rathi [28]	2002	USA	56.0	20	29	6	33	MSP	BOT
Yoon [21]	2001	USA	NA	8	12	0	10	BSP	NT

BOT: benign ovarian tissues AT: adjacent tissues; NT: normal ovarian tissues of cancer-free patients or healthy people; NA: not available; MSP: methylation-specific polymerase chain reaction; MS-MLPA: methylation-specific multiplex ligation-dependent probe amplification; BSP: bisulfite sequencing PCR; M: *RASSF1A* methylated; U: *RASSF1A* unmethylated.

The results of the NOS showed that most studies had a hospital control except for Li [26] and had not matched the control according to the potential confounder except for Ibanez [20]. The score of the studies ranged from 5 to 8 with a median score of 7. Detail information of the NOS was shown in Table 2.

**Table 2:** The quality assessment of included studies.

	Newcastle-Ottawa Scale*									
Author	1	2	3	4	5A	5B	6	7	8	Total
Ho [22]	Yes	Yes	No	Yes	Yes	No	Yes	Yes	Yes	7
Montavon [16]	Yes	Yes	No	Yes	Yes	No	Yes	Yes	Yes	7
Bhagat [23]	Yes	Yes	No	Yes	Yes	No	Yes	Yes	Yes	7
He [24]	Yes	Yes	No	Yes	Yes	No	Yes	Yes	Yes	7
Chen [17]	Yes	Yes	No	Yes	Yes	No	Yes	Yes	Yes	7
Shen [25]	Yes	Yes	No	No	Yes	No	Yes	Yes	Yes	6
Wu [18]	Yes	Yes	No	Yes	No	No	Yes	Yes	Yes	5
Li [26]	Yes	Yes	Yes	Yes	Yes	No	Yes	Yes	Yes	8
Makarla [27]	Yes	Yes	No	Yes	Yes	No	Yes	Yes	Yes	7
Ma [19]	Yes	No	No	Yes	Yes	No	Yes	Yes	Yes	5
Ibanez [20]	Yes	Yes	No	Yes	Yes	Yes	Yes	Yes	Yes	8
Rathi [28]	Yes	Yes	No	Yes	Yes	No	Yes	Yes	Yes	7
Yoon [21]	Yes	Yes	No	Yes	Yes	No	Yes	Yes	Yes	7

* 1 indicates case definition and diagnostic appropriate; 2, consecutive patients or case having a good representation; 3, community controls; 4, control with no the history of study disease; 5A, according to the most important factor to select and analyze control; 5B, according to the second important factor to select and analyze control; 6, ascertainment of exposure by blinded interview or record; 7, same method of ascertainment used for cases and controls; and 8, non-response rate the same for cases and controls.

### Meta-analysis

**Figure 2:**
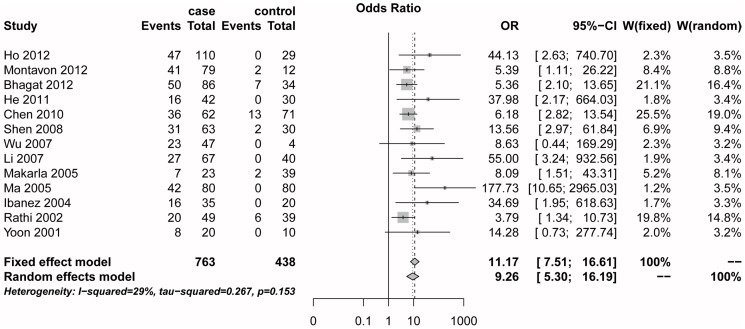
The estimates for *RASSF1A* methylation frequency associated with ovarian cancer in the meta-analysis.

The *x* ^2^-based Cochran Q statistic test and *I* ^2^ statistics did not detect a significant heterogeneity among the included studies (*I^2^* = 29.0%, Q = 16.9, *P = *0.15). Then, we used a fixed-effects model to assess the association between *RASSF1A* promoter methylation and ovarian cancer. In the meta-analysis, *RASSF1A* promoter methylation frequency was significantly associated with ovarian cancer (Summary OR was 11.17, 95% CI = 7.51–16.61) (Fig. 2).

### Meta-regression and Subgroup Analysis

Although tests did not find a significant heterogeneity across the studies, we first conducted meta-regression to explore potential sources of heterogeneity. Based on previous studies we assumed that heterogeneity may come from the population subgroup, sample size and control sample type. We conducted a multiple regression model with three variables (i.e. population subgroup, publication year, and case sample size). In the end, no source of significant heterogeneity was found (Table 3). We performed a subgroup analysis to further evaluate the source of the heterogeneity according to populations, case sample size and control type.

**Table 3:** Meta-regression analysis.

		95% CI		
Heterogeneity sources	Coefficient	Lower	Upper	*P*
Population	−1.10	−2.91	0.71	0.23
Publication year	−0.09	−0.34	0.16	0.48
Case sample size	−0.12	−2.39	2.16	0.92

In the subgroup analysis, the OR between the *RASSF1A* promoter methylation and ovarian cancer was 14.76 (95% CI = 5.73–38.01) in Asians and 6.85 (95% CI = 3.46–13.58) in Caucasians under the fixed-effects model. Similarly, the OR for the case sample size subgroup was 8.93 (95% CI = 4.43–18.42) in the <50 case group under the fixed-effects model, and 11.19 (95% CI = 4.83–25.96) in the ≥50 case group under the random-effects model. In the subgroup analysis of control type, the OR was 8.43 (95% CI = 4.03–17.63) in the BAT group including benign ovarian tissues and adjacent tissues under the random-effects model and 16.50 (95% CI = 6.82–39.88) in the NT group under the fixed-effects model (Table 4).

**Table 4:** Subgroup analysis of the association between *RASSF1A* promoter methylation and ovarian cancer.

	Cancer		Normal		M-H pooled OR†	D+L pooled OR‡	Heterogeneity		
Group	M+	N	M+	N	OR (95% CI)	OR (95% CI)	*I^2^* (%)	*P*	τ^2^
Total	364	763	32	438	11.17 (7.51–16.61)	9.26 (5.30–16.19)	29.0	0.153	0.27
Population subgroup									
Asians	249	510	22	314	13.97 (8.54–22.85)	14.76 (5.73–38.01)	55.0	0.038	0.74
Caucasians	115	253	10	124	6.85 (3.46–13.58)	5.97 (2.96–12.05)	0.0	0.724	<0.01
Case sample size									
<50	90	216	8	142	8.93 (4.33–18.42)	6.96 (3.27–14.81)	0	0.473	<0.01
≥50	243	547	24	296	12.26 (7.62–19.72)	11.19 (4.83–25.96)	51.3	0.055	0.57
Control type									
BAT	356	743	29	336	8.95 (5.91–13.55)	8.43 (4.03–17.63)	48.3	0.031	0.67
NT	164	331	3	102	16.50 (6.82–39.88)	12.82 (5.08–32.37)	0.0	0.566	<0.01

BAT: benign ovarian tissues and adjacent tissues; NT: normal ovarian tissues of cancer-free patients or healthy people.

† the fixed-effects model.

‡ the random-effects model.

### Sensitivity Analysis

**Figure 3:**
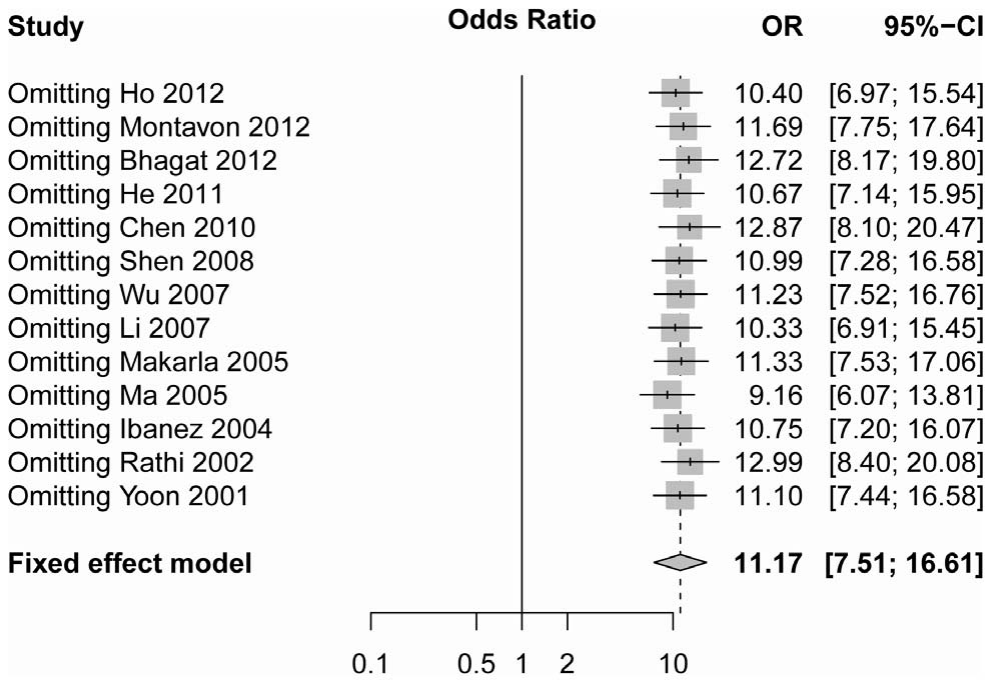
The sensitivity analysis by omitting a single study under the fixed-effects method.

According to sensitivity analysis the odds ratio ranged from 9.16 (95% CI = 6.07–13.81) to 12.99 (95% CI = 8.40–20.08) by omitting a single study under the random-effect model (Fig. 3). No single study was found to affect the pooled OR as indicated by sensitivity analysis.

### Publication Bias

**Figure 4:**
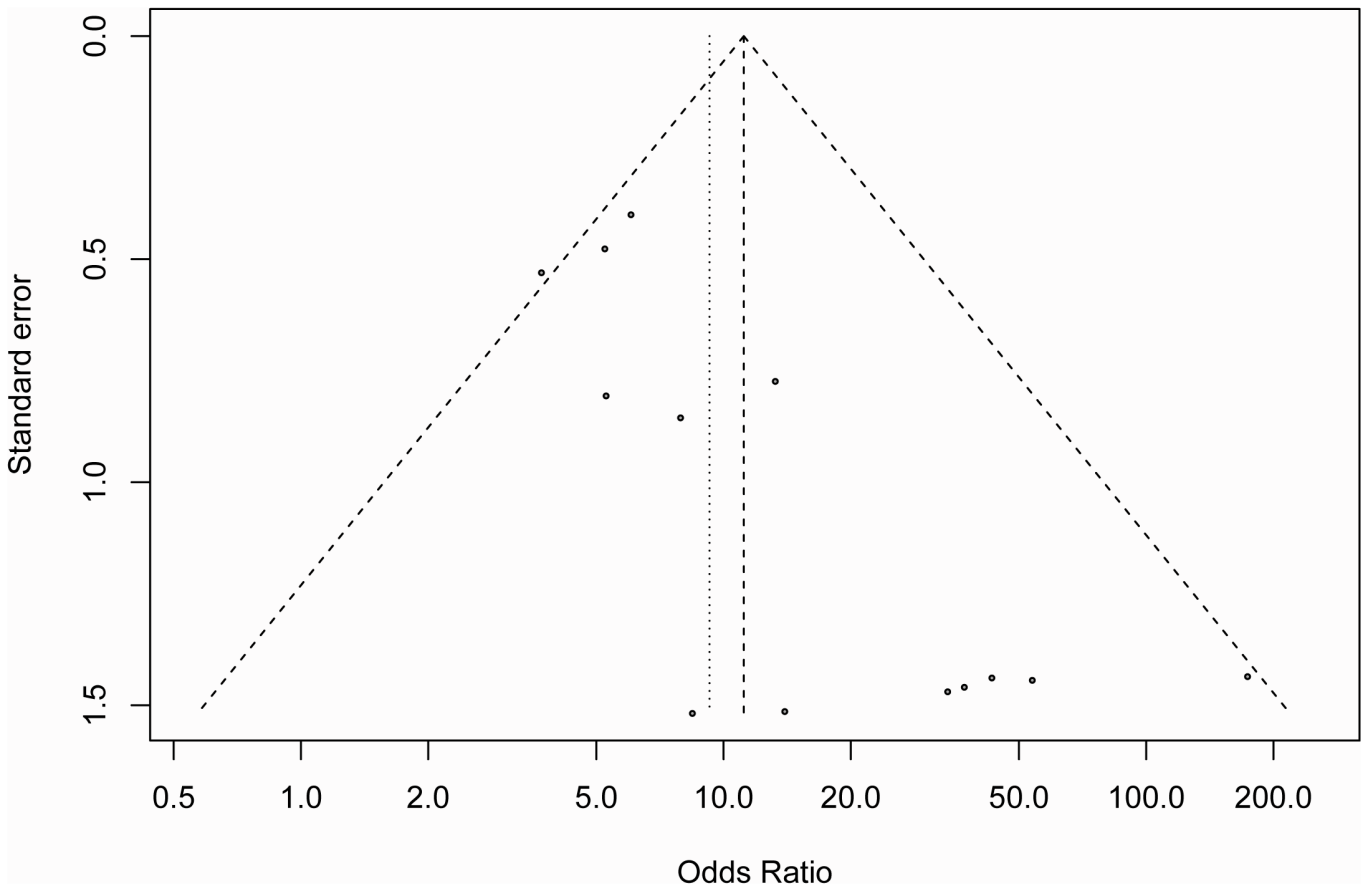
The Begg’s funnel plot for assessment of publication bias in the meta-analysis (each study is represented by an point).

The study by Peters [29] showed that type I error rates for the Egger’s test [13] are higher than those for the alternative regression test when the summary estimates are lnORs. Therefore, Begg’s rank correlation, a funnel plot and the Peters test were employed to assess the publication bias of the literature. The shape of the Begg’s funnel plot in Figure 4 showed a possible asymmetry but the Peters test did not detect publication bias (*P = *0.48) and the Begg’s rank correlation also did not detect publication bias (*P = *0.14). The fail-safe number (*Z* = 48.23, N_fs0.05_ = 851.98, N_fs0.01_ = 415.53) also suggested that the study had a very small degree of publication bias.

## Discussion

*RASSF1A* modulates multiple apoptotic, tubulin dynamics and cell cycle checkpoint pathways. Some studies found that the overexpression of *RASSF1A* promotes apoptosis and cell cycle arrest in cancer cell lines [9], [30]. *RASSF1A* can be inactivated by gene mutation and promoter methylation and the latter accounted for the vast majority of cases [9]. *RASSF1A* promoter methylation is one of the most frequent epigenetic inactivation events detected in human cancer and leads to silencing of *RASSF1A* expression [31]. Loss of *RASSF1A* expression has been reported in lung, breast, bladder, gastric, cholangiocarcinoma and oesophageal sqaumous cell carcinoma primary tumors [32]–[36].

The present meta-analysis included thirteen articles with 763 cases and 438 controls. The *RASSF1A* methylation level of the cancer group was significantly higher than the control group. The pooled odds ratio under fixed-effect model was 11.17 (95% CI = 7.51–16.61) in the cancer cases versus the controls. The result demonstrated that *RASSF1A* promoter methylation was associated with ovarian cancer, which was consistent with previous studies [22], [23].

The summary OR was 14.76 (95% CI = 5.73–38.01) in Asians and 6.85 (95% CI = 3.46–13.58) in Caucasians. The association between *RASSF1A* promoter methylation and ovarian cancer in Asians was stronger than that in Caucasians. Similarly,Fraser’s study observed divergence of DNA methylation between African and European population, which may be due to methylation-associated SNPs (mSNPs) and complex epistasis or gene × environment interactions [37].

For sample size, the summary OR was 8.93 (95% CI = 4.43–18.42) in the <50 case group and 11.19 (95% CI = 4.83–25.96) in the ≥50 case group. In another subgroup the summary OR changed a little between <50 case group and ≥50 case group. The ORs for the different control subgroup were 8.43 (95% CI = 4.03–17.63) for the BAT group and 16.50 (95% CI = 6.82–39.88) for the NT group. This result showed that the difference in frequency of *RASSF1A* promoter methylation between the case group and the NT group was greater than that between the case group and the BAT group. The result indicated that benign ovarian tissues and adjacent tissues had a higher frequency of *RASSF1A* promoter methylation than normal ovarian tissue, which also suggested that *RASSF1A* promoter methylation may play an important role in the pathogenesis of ovarian carcinoma [23], [38].

There were some limitations in the study. The first limitation is potential confounder. Because there was a lack of information about variables in the control group, we only considered three variables (population, case sample size, and control type) in the subgroup analysis. Another possible confounding factors could exist in the meta-analysis. The second limitation is publication bias. Although no significant publication bias was found according to the Peter’s test, negative and unpublished studies may lead to some bias.

In conclusion, hypermethylation of *RASSF1A* promoter was found to be associated with ovarian cancer according to the meta-analysis, which suggested that the promoter methylation of *RASSF1A* is a potentially useful biomarker in the carcinogenic process of ovarian cancer. The present study requires confirmation through well-designed prospective studies.

## Supporting Information

Checklist S1 PRISMA Checklist.
(DOCX)
